# Spontaneous late-onset pupillary block in a pseudophakic, vitrectomized eye despite prior Nd:YAG capsulotomy: a diagnostic challenge

**DOI:** 10.22336/rjo.2026.46

**Published:** 2026

**Authors:** Batuhan Aksoy, Cansu Yüksel Elgin1

**Affiliations:** 1 Ophthalmology Department, Istanbul University-Cerrahpasa Medical Faculty, Istanbul, Turkey

**Keywords:** pupillary block, pseudophakia, laser peripheral iridotomy, acute angle closure, YAG capsulotomy, IOP = Intraocular pressure, OCT = Optical coherence tomography, UBM = Ultrasound biomicroscopy, YAG = Yttrium-Aluminum-Garnet (laser), LPI = Laser peripheral iridotomy, POAG = Primary open-angle glaucoma, PPV = Pars plana vitrectomy, PCIOL = Posterior chamber intraocular lens, OD = Oculus dexter (right eye), OS = Oculus sinister (left eye)

## Abstract

**Objective:**

To report a rare case of spontaneous, delayed-onset pupillary block in a pseudophakic, vitrectomized eye with a prior Nd: YAG posterior capsulotomy, and to emphasize the importance of maintaining a high index of suspicion even in anatomically protected eyes.

**Methods:**

We describe the clinical presentation, imaging findings, differential diagnosis, and management of a 78-year-old female with well-controlled primary open-angle glaucoma who presented with acute angle-closure symptoms in a previously vitrectomized, pseudophakic eye. Multimodal imaging, including anterior segment optical coherence tomography (OCT) and ultrasound biomicroscopy (UBM), was utilized to rule out malignant glaucoma and aqueous misdirection.

**Results:**

Slit-lamp examination revealed iris bombe and appositional angle closure in the left eye. Anterior segment OCT confirmed a shallow anterior chamber, while UBM excluded ciliochoroidal effusion and anterior rotation of the ciliary body. A diagnosis of acute pupillary block was established. Nd: YAG laser peripheral iridotomy (LPI) resulted in immediate resolution of symptoms and normalization of intraocular pressure.

**Discussion:**

Although pupillary block is generally considered unlikely in pseudophakic and vitrectomized eyes, this case demonstrates that it may still occur despite prior Nd:YAG posterior capsulotomy. Progressive anatomical changes may contribute to delayed obstruction of aqueous flow. Careful clinical examination, combined with anterior segment OCT and UBM, is essential for distinguishing pupillary block from other causes of secondary angle closure, particularly malignant glaucoma and aqueous misdirection.

**Conclusions:**

This case demonstrates that pupillary block can occur spontaneously even in eyes with prior vitrectomy and YAG capsulotomy. Progressive anatomical changes may lead to delayed iris-lens apposition. Early diagnosis and treatment with LPI can reverse the condition and preserve vision. Clinicians should consider pupillary block in the differential diagnosis of acute angle closure, regardless of prior surgical history.

## Introduction

Pupillary block is a well-established mechanism of secondary angle closure, most commonly observed in phakic eyes or shortly after cataract surgery. It may also occur in association with intraocular inflammation, such as uveitis. In pseudophakic eyes that have undergone pars plana vitrectomy (PPV), the risk of pupillary block is generally considered low due to disruption of the anterior hyaloid and altered vitreous dynamics, which facilitate aqueous humor flow. Additionally, Nd: YAG laser posterior capsulotomy further reduces resistance between the posterior and anterior chambers, thereby decreasing the likelihood of block formation [1]. Nevertheless, pupillary block can still occur under rare circumstances.

We report a unique case of acute pupillary block glaucoma occurring five years after combined phacoemulsification and PPV in a pseudophakic, vitrectomized patient with prior YAG capsulotomy. This case is particularly noteworthy due to the absence of identifiable predisposing factors, suggesting a truly spontaneous onset. Written informed consent was obtained from the patient for the publication of this case report and accompanying images.

## Case presentation

A 78-year-old woman with a 15-year history of well-controlled primary open-angle glaucoma (POAG) presented to the emergency department with sudden-onset severe ocular pain, brow headache, nausea, and vomiting in the left eye. Her ophthalmic history included combined phacoemulsification, PPV, and posterior chamber intraocular lens (PCIOL) implantation performed five years earlier for a full-thickness macular hole. Two years postoperatively, she underwent bilateral Nd: YAG posterior capsulotomy for posterior capsule opacification. Her glaucoma regimen included fixed-dose dorzolamide-timolol and bimatoprost.

**Fig. 1A:**
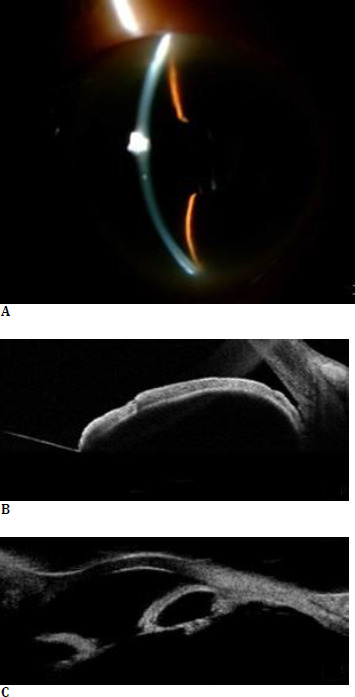
Slit-lamp photograph demonstrating pupillary block in a pseudophakic patient, characterized by a classic iris bombe configuration; B. Anterior segment optical coherence tomography (AS-OCT) image demonstrating closure of the iridocorneal angle of the left eye; C. Ultrasound biomicroscopy (UBM) image demonstrating an iris bombe configuration, with the ciliary body in normal position and no evidence of ciliochoroidal effusion

On presentation, intraocular pressure (IOP) was measured at 16 mmHg in the right eye (OD) and 70 mmHg in the left eye (OS). Intravenous 20% mannitol (5 cc/kg) was administered, reducing IOP to 37 mmHg within 30 minutes. Additional topical hypotensive agents were administered. Best-corrected visual acuity was 0.10 logMAR (OD) and 0.40 logMAR (OS). Slit-lamp examination revealed a pseudophakic eye with prior YAG capsulotomy and iris bombé configuration in the left eye, without signs of anterior chamber inflammation (Fig. 1A). Fundus examination showed non-exudative age-related macular degeneration in both eyes and healthy optic nerves with a cup-to-disc ratio of 0.2 bilaterally.

Gonioscopy revealed 360° appositional angle closure in the left eye. Anterior segment optical coherence tomography (OCT) confirmed a shallow anterior chamber with angle narrowing (Fig. 1B). Ultrasound biomicroscopy (UBM) excluded ciliochoroidal effusion and malignant glaucoma by demonstrating no anterior rotation of the ciliary body (Fig. 1C). Based on the absence of aqueous misdirection, normal posterior segment anatomy, and the presence of iris bombé, a diagnosis of acute pupillary block was made.

**Fig. 2A:**
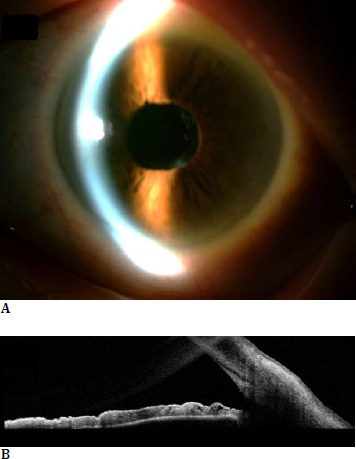
Slit-lamp photograph of the pseudophakic left eye with prior Nd: YAG laser capsulotomy, demonstrating resolution of the iris bombe configuration following Nd: YAG laser iridotomy, with a patent iridotomy site; B. Anterior segment optical coherence tomography (AS-OCT) image demonstrating widening of the iridocorneal angle following Nd: YAG laser iridotomy

A Nd: YAG laser peripheral iridotomy (LPI) was performed, resulting in immediate normalization of IOP to 14 mmHg in both eyes and resolution of the iris bombé (Fig. 2A). Post-procedure anterior segment OCT confirmed deepening of the anterior chamber and restoration of aqueous flow (Fig. 2B).

## Discussion

This case challenges the conventional understanding that pseudophakia, vitrectomy, and YAG capsulotomy collectively protect against pupillary block. While pupillary block is typically associated with a sealed posterior capsule or phakic status, a few cases have reported this mechanism in pseudophakic eyes, mostly in the early postoperative period or in association with intraocular inflammation or anatomical abnormalities [2]. Vitreous block can occur in pseudophakic patients, especially with a ruptured posterior capsule, often requiring anterior vitrectomy and iridectomy. General anesthesia may also trigger vitreous block in susceptible cases. However, in this case, prior pars plana vitrectomy for epiretinal membrane and a patent YAG capsulotomy for posterior capsule opacification excluded vitreous block as the cause [3].

Our patient’s presentation was remarkable for its delayed onset—occurring five years after combined phaco-vitrectomy—and the absence of any inciting factor such as surgery, trauma, or medications known to induce ciliochoroidal effusion. Furthermore, multimodal imaging ruled out aqueous misdirection and other secondary causes of angle closure. The presence of iris bombé, confirmed by slit-lamp biomicroscopy and anterior segment OCT, was pathognomonic for pupillary block, while UBM provided crucial exclusion of posterior segment pathology [4].

Several hypotheses may explain late-onset pupillary block in such eyes. Progressive zonular weakness, subclinical synechiae formation, fibrotic membrane development, or anterior capsular contraction may alter aqueous humor dynamics, promoting iris-lens apposition and leading to block formation. In capsular block syndrome, intraocular pressure can be elevated in both early and late postoperative periods, and Nd:YAG capsulotomy is commonly used as a treatment; however, in this case, an Nd:YAG capsulotomy had already been performed years earlier. These changes may occur insidiously over time, resulting in spontaneous onset in the absence of overt pathology [1,5].

The prompt resolution of symptoms and normalization of IOP following LPI underscore the reversibility of this condition when identified promptly. Additionally, the effectiveness and safety of LPI have been well documented in the literature, with studies supporting its use as a reliable treatment option with no significant adverse effects over long-term follow-up [6].

Although cases of pseudophakic pupillary block are generally reported in the early postoperative period in the literature, late-onset pupillary block occurring years after YAG capsulotomy, a treatment for this condition, has not yet been reported. Distinguishing between pupillary block and malignant glaucoma is critical, as their management differs significantly. The exact mechanism resulting in aqueous misdirection is unknown. Still, it appears to be related to the relationship between the ciliary body, anterior hyaloid, and lens, as well as the permeability of the vitreous [7]. Multimodal imaging, particularly anterior segment OCT and UBM, plays a pivotal role in accurate diagnosis [8].

## Conclusion

Although rare in pseudophakic and vitrectomized eyes with prior YAG capsulotomy, pupillary block should remain on the differential diagnosis in cases of acute angle closure. This case illustrates that such a block can arise spontaneously, without identifiable external triggers. Early recognition through clinical examination and imaging, followed by timely LPI, can lead to complete resolution and visual recovery. Clinicians must remain vigilant for this uncommon yet reversible cause of secondary angle closure, even in anatomically protected eyes. The presence of a patent YAG capsulotomy, which typically facilitates aqueous flow and reduces the risk of aqueous misdirection, does not fully exclude the possibility of pupillary block in the long term.
